# Manual Dexterity Rehabilitation in Parkinson’s Disease and Paranoid Schizophrenia: A Controlled Study

**DOI:** 10.3390/life16020196

**Published:** 2026-01-24

**Authors:** Tatiana Balint, Alina-Mihaela Cristuta, Adina Camelia Slicaru, Ilie Onu, Daniel Andrei Iordan, Ana Onu

**Affiliations:** 1 Faculty of Movement, Sports, and Health Sciences, “Vasile Alecsandri” University of Bacău, 600115 Bacau, Romania; tbalint@ub.ro (T.B.); cristuta.alina@ub.ro (A.-M.C.); slicaruadinacamelia@ub.ro (A.C.S.); 2 Department of Biomedical Sciences, Grigore T. Popa University of Medicine and Pharmacy Iasi, 700454 Iasi, Romania; 3 Department of Physical Therapy, Elipetro Med Clinic, 610119 Piatra Neamt, Romania; 4 Center of Physical Therapy, Rehabilitation and Wellness, “Dunărea de Jos” University of Galati, 800008 Galati, Romania; 5 Department of Individual Sports and Kinetotherapy, Faculty of Physical Education and Sport, “Dunărea de Jos” University of Galati, 800008 Galati, Romania; 6 Doctoral School, Grigore T. Popa University of Medicine and Pharmacy Iasi, 700115 Iasi, Romania; chirila.ana@d.umfiasi.ro

**Keywords:** manual dexterity, Parkinson’s disease, paranoid schizophrenia, physiotherapy, fine motor control, upper limb rehabilitation, Coin Rotation Task, Purdue Pegboard Test, Kapandji scale

## Abstract

Background: Manual dexterity (MD) impairment is a frequent and disabling feature in patients with Parkinson’s disease (PD) and paranoid schizophrenia (PS), significantly affecting functional independence and activities of daily living. However, rehabilitation strategies specifically targeting fine motor control remain insufficiently integrated into routine physiotherapy (PT). Objective: This study investigated the effects of a structured, progressive PT program incorporating targeted MD training on upper limb function in patients with PD and PS. Methods: A prospective, exploratory, interventional study was conducted in 30 patients, allocated to either an experimental group (EG, *n* = 20) or a control group (CG, *n* = 10). Participants had PD (Hoehn and Yahr stages II–III) or chronic, clinically stable PS. MD was assessed using the Purdue Pegboard Test, Coin Rotation Task, and Kapandji opposition score. The EG completed a four-phase, 40-week dexterity-oriented rehabilitation program, while the CG received standard disease-specific PT. Between-group differences in change scores were analyzed using one-way ANOVA. Results: The EG showed significantly greater improvements than the CG in thumb opposition, psychomotor processing speed, and unilateral and bilateral fine motor performance (*p* < 0.001 for all), with large to very large effect sizes (η^2^ = 0.45–0.76). No significant between-group differences were observed for complex sequential assembly tasks. Conclusions: Integrating targeted MD training into structured PT programs significantly improves fine motor performance in patients with PD and PS, supporting its inclusion in rehabilitation protocols for residential and outpatient care settings.

## 1. Introduction

Manual dexterity (MD) represents a highly complex motor behavior characterized by precision, adaptability, and fine coordination of the upper limb segments. It relies on the integrated functioning of cortical and subcortical motor control, peripheral neural transmission, musculoskeletal integrity, and continuous sensory feedback [1]. Even apparently simple actions, such as grasping an object, require anticipatory hand shaping according to the object’s size and geometry, along with coordinated movements of multiple finger joints and a distinct functional independence of the thumb, highlighting the sophistication of human hand motor control [2].

Across the lifespan, MD undergoes progressive decline due to physiological, neurological, and cognitive changes associated with aging [3]. Reductions in muscle strength, joint mobility, nerve conduction velocity, and executive functioning negatively affect fine motor control and bimanual coordination, with direct consequences for functional independence [4]. Importantly, recent evidence suggests that MD is closely linked to higher-order cognitive functions, particularly executive control, making fine motor performance a potential functional marker of global cognitive status in older adults [5].

At a global level, disability remains a major public health challenge. According to the World Report on Disability, approximately 15% of the world’s population lives with some form of disability, with 2–4% experiencing significant functional limitations [6]. In Romania, official data indicate that over 930,000 individuals were registered with disabilities in 2024, with the vast majority living in family settings or independently, but a substantial proportion residing in institutional care facilities [7].

Demographic aging, declining birth rates, and the increasing prevalence of chronic neurological, oncological, and cardiovascular conditions have led to a growing population of older adults with complex functional impairments [8,9]. In this context, the Romanian healthcare system faces significant challenges in delivering adequate long-term and rehabilitative care, particularly for individuals with irreversible or progressive conditions [10].

Institutionalized populations are especially vulnerable to accelerated physical and cognitive decline due to reduced environmental stimulation, limited engagement in meaningful activities, and insufficient access to structured rehabilitation programs [11,12,13,14].

Within residential care settings, the preservation and enhancement of MD play a critical therapeutic role [15]. Fine motor abilities have a direct influence on autonomy in activities of daily living (ADL), social participation, and perceived dignity [15,16]. Moreover, improving MD may reduce caregiver burden by increasing residents’ self-sufficiency, allowing healthcare staff to allocate resources more efficiently toward complex care needs. Despite its functional relevance, MD is often underassessed and insufficiently targeted in routine rehabilitation programs for individuals residing in institutions [17,18,19].

Therefore, the evaluation and rehabilitation of MD from a physiotherapeutic (PT) perspective represent a timely and clinically relevant research topic. A structured assessment combined with individualized PT interventions may support the maintenance or recovery of fine motor skills, thereby enhancing functional independence and quality of life in institutionalized populations [20,21,22].

Prehension represents the biomechanical and sensorimotor expression of MD, as the precise grasping and manipulation of objects emerge from the coordinated interaction between fine motor control, sensory feedback, and intersegmental upper limb coordination [15]. Prehension constitutes a fundamental function of the upper limb and a key determinant of human interaction with the environment. The hand, as the terminal segment of the upper limb, is highly specialized for both fine and forceful movements, with its functionality emerging from the integrated action of osteoarticular structures, the musculotendinous system, and sensory feedback mechanisms [18,22,23].

Upper limb functionality can be markedly compromised in pathological contexts. Traumatic musculoskeletal injuries may impair joint stability, range of motion (ROM), and intersegmental coordination, while peripheral nerve lesions affecting the median, ulnar, or radial nerves result in combined motor and sensory deficits, leading to reduced fine prehension and MD. Central nervous system disorders exert an even more profound impact by disrupting motor planning, sensorimotor integration, and force modulation [24,,25,26,27,28].

Parkinson’s disease (PD) is characterized by degeneration of dopaminergic neurons in the substantia nigra, leading to basal ganglia dysfunction and clinically manifesting as bradykinesia, rigidity, resting tremor, and postural instability, all of which compromise grip stability and movement execution [21,29,30]. Similarly, stroke frequently results in hemiparesis, spasticity, sensory deficits, and apraxia, limiting grasping ability, object manipulation, and coordinated intersegmental movements [31,32,33,34,35].

Beyond classical neurological disorders, psychiatric conditions may also impair MD. Paranoid schizophrenia (PS) has been associated with clinically relevant deficits in grip force regulation, temporal coordination of finger movements, and interdependence of finger actions, reflecting dysfunction within cortico-subcortical motor circuits and interfering with precise prehension [36,37].

Based on the present findings, the following hypotheses are proposed to guide future clinical practice and research in the rehabilitation of MD in PD and PS:

**H1.**  *A structured physiotherapy program incorporating targeted MD training produces greater improvements in upper limb function than standard PT in patients with PD and PS.*

**H2.**  *MD training leads to significant gains in thumb opposition, psychomotor processing speed, and unilateral and bilateral fine motor performance.*

**H3.**  *Improvements are more pronounced in simple and coordinated fine motor tasks than in complex sequential assembly tasks.*

**H4.**  *Standard PT without dexterity-focused components is insufficient to achieve meaningful improvements in MD in these patient populations.*

## 2. Materials and Methods

### 2.1. Study Design

This study was designed as a prospective, exploratory, interventional study to evaluate the effects of a structured and individualized PT program on MD in patients diagnosed with PD and PS. The research was conducted between August 2024 and August 2025, following a phased methodological framework that integrated functional assessment, therapeutic intervention, and analysis of results.

The study was conducted within the Physiotherapy Department of the Elipetro Med Clinic in Piatra Neamț, with all examinations and data collection procedures being performed by authorized personnel. All procedures were performed in accordance with the principles outlined in the Declaration of Helsinki. To ensure the reproducibility of the assessment and methodological consistency, all assessments were performed under identical conditions by the same neurologist and physiotherapist, both of whom are members of the multidisciplinary assessment team.

The study protocol was approved by the Elipetro Med Scientific Research Ethics Committee, and written informed consent was obtained from all participants before their inclusion.

### 2.2. Participants

A total of 30 patients were enrolled in the study and allocated to either the experimental group (EG, *n* = 20) or the control group (CG, *n* = 10). All participants had a clinically confirmed diagnosis of Parkinson’s disease (PD; Hoehn and Yahr stages II–III) or Parkinsonian syndrome (PS), were in a chronic and clinically stable stage, and presented with impaired manual dexterity. Both male and female patients were included, with a comparable sex distribution between groups. Dementia and dyslipidemia were recorded as associated conditions at baseline. Written informed consent was obtained from all participants before inclusion.

Group allocation followed a pragmatic, non-randomized approach based on clinical feasibility and availability within the rehabilitation setting, in line with the exploratory design of the study. Participants were assigned to the EG or CG to achieve comparable baseline functional status and diagnostic representation. Consequently, both groups included patients with PD (Hoehn and Yahr stages II–III) and chronic, clinically stable PS, with no relevant differences observed between groups regarding age, sex distribution, diagnosis, or baseline MD.

Eligible participants were older adults (mean age ≈ 75 years), reflecting the demographic profile commonly associated with advanced neurological and psychiatric conditions and increased vulnerability to functional decline. This age range was considered appropriate, as fine motor impairments tend to become more pronounced with advancing age and disease progression.

Inclusion criteria comprised the ability to provide informed consent (or consent provided by a legal representative), a minimum cognitive functioning level allowing participation in assessments and PT, a clinically confirmed diagnosis, clinical stability over the previous 6 months, and availability to attend regular PT sessions. Exclusion criteria included other major neurological conditions (e.g., stroke, epilepsy), severe psychiatric disorders, severe medical comorbidities, severe cognitive impairment, participation in other clinical studies, contraindications to study procedures, or recent major changes in pharmacological treatment.

The EG received a structured and individualized rehabilitation program incorporating targeted MD training, while the CG followed standard disease-specific PT without focused fine motor interventions. This design enabled a comparative evaluation of dexterity-oriented rehabilitation versus conventional PT.

All participants were clinically stable for at least six months before inclusion and continued their usual pharmacological treatment throughout the study. PD patients were assessed in their usual medication “on” state, while patients with PS received stable antipsychotic treatment. Detailed stratification by medication class, dose, or extrapyramidal symptom severity was not systematically performed.

### 2.3. Evaluation

Functional assessment was performed at baseline and repeatedly during the intervention using standardized, validated instruments combined with clinical observation. Data were recorded in individualized clinico-functional assessment files.

MD was primarily evaluated using the Purdue Pegboard Test (PPT), Coin Rotation Task (CRT), and Kapandji scale.

The PPT is a standardized measure of MD and bimanual coordination that assesses the speed and accuracy of fine motor performance and is widely used in neurological and psychiatric populations to monitor functional changes following rehabilitation. The apparatus consists of a board with two vertical rows of holes and compartments containing pegs, collars, and washers [38]. The PPT includes four timed subtests: right hand (30 s), left hand (30 s), both hands (30 s), and assembly (60 s). Scores correspond to the number of pegs inserted or assemblies completed within the allotted time, with higher values indicating better dexterity and coordination [38]. In this study, PPT scores were interpreted relative to baseline performance and between-group differences rather than normative values, given the advanced age and neurological/psychiatric profile of the participants [39,40].

The CRT was used to assess psychomotor processing speed and fine digital control by counting the number of complete 180° coin rotations performed within 10 s, following predefined validity and exclusion criteria. Performance was scaled as the total number of rotations achieved, with higher scores reflecting superior psychomotor speed and MD [41]. In conditions such as PD and PS, impaired psychomotor speed and reduced MD are common and clinically relevant. Previous studies have demonstrated good convergent and discriminant validity of the CRT, minimal influence of demographic factors, and adequate sensitivity and specificity for distinguishing mild motor impairment from healthy performance [42], supporting its clinical utility for detecting subtle deficits and monitoring functional change [43,44].

Additional assessments included systematic clinical observation of prehension patterns, with emphasis on power and precision grips, frequently altered in PD and PS due to bradykinesia, rigidity, or psychomotor slowing. Thumb opposition was quantitatively assessed using the Kapandji scale, providing an objective measure of thenar function and opposition range, which may be compromised in Parkinsonian syndromes and schizophrenia-related fine motor dysfunction [45,46,47]. Performance was graded using predefined ordinal and qualitative scales ranging from 0 to 10, enabling stratification of MD impairment severity and comparison across assessment time points [45].

### 2.4. Rehabilitation Program

Participants allocated to the EG underwent a structured, progressive PT program specifically designed to maintain and improve MD within a broader functional rehabilitation framework. The intervention addressed both proximal and distal segments of the upper limb and was adapted to disease-specific motor, sensory, and cognitive characteristics. The total duration of the program was 40 weeks, divided into four consecutive phases of 10 weeks each. Sessions were conducted three times per week, with a standardized duration of 30 min per session. Progression was individualized based on patient tolerance, performance quality, and fatigue levels.

#### 2.4.1. Phase I—Joint Mobilization and Segment Preparation (Weeks 1–10)

The primary objectives of the initial phase were to reduce rigidity, increase joint ROM, and enhance sensory awareness. Interventions included passive, assisted, and active mobilization of the wrist, hand, and finger joints, complemented by stretching exercises maintained for approximately 20 s. Basic grip activities using soft and compliant objects were introduced to promote safe engagement and early functional activation.

PNF techniques such as rhythmic initiation, rhythmic rotation, slow reversal, and sequential strengthening were systematically applied to facilitate movement initiation, improve neuromuscular coordination, and enhance proprioceptive feedback. Exercises were supported by visual monitoring of movement and clear, rhythmic verbal cues to sustain attention and motivation. This phase established a stable motor foundation and reduced segmental limitations, preparing participants for more demanding functional tasks.

#### 2.4.2. Phase II—Muscle Strengthening and Prehension Training (Weeks 11–20)

The second phase focused on progressive strengthening of intrinsic and extrinsic hand muscles and the re-education of prehension patterns. Therapeutic activities included object grasping, transfer tasks, and precision pinch exercises using small devices such as balls, cylinders, and sponges with varying resistance and texture.

PNF techniques (slow reversal, slow reversal with opposition, and agonistic reversals) were employed for flexor–extensor, pronator–supinator, and radial–ulnar deviation movements at the wrist and hand joints. These techniques aimed to optimize voluntary motor response, improve agonist–antagonist coordination, and enhance kinesthetic perception. Both gross grasp patterns (polydigitopalmar, digitopalmar) and fine prehension patterns (bidigital, tridigital, terminal, and lateral grips) were systematically trained, with gradual increases in task complexity and resistance.

#### 2.4.3. Phase III—Fine Motor Control and Thumb Opposition Re-Education (Weeks 21–30)

The third phase emphasized fine motor control, coordinated finger movements, and thumb opposition, which are critical for effective hand function. Task-oriented activities involved the manipulation of small objects, sequential finger movements, and precision tasks adapted to daily living requirements.

Given the central biomechanical and functional role of the thumb in prehension, specific exercises targeted thumb opposition, stabilization, and dissociation from adjacent fingers. Movement execution was paced using verbal and visual cues to counteract bradykinesia and enhance movement timing and accuracy. This phase aimed to refine movement quality, improve functional precision, and support motor learning through repetitive, meaningful tasks.

#### 2.4.4. Phase IV—Functional Integration and Progress Monitoring (Weeks 31–40)

The final phase focused on transferring acquired motor skills into functional ADLs, such as buttoning, writing, utensil handling, and simulated household tasks. PPT and CRT tasks were also incorporated as therapeutic exercises to reinforce fine motor performance under functional conditions.

Exercises progressed from isolated movements to complex, multi-step activities requiring coordination, sequencing, and sustained attention. Global movements of the upper limb and trunk were included to promote psychomotor integration and postural control. Each session concluded with breathing exercises and structured feedback on performance and progress, reinforcing patient engagement and adherence.

Participants allocated to the CG received standard disease-specific PT comparable in frequency, duration, and overall therapeutic exposure to the experimental intervention, to minimize potential dose-related bias. The program was delivered over 40 weeks, with three sessions per week, each lasting approximately 30 min, consistent with routine clinical practice.

The control intervention focused on global motor function and general upper limb use, aiming to maintain joint mobility, muscle activation, postural control, and functional independence. Therapy included active and assisted ROM exercises for the upper limbs, general strengthening and endurance exercises involving the shoulder, elbow, and wrist, as well as postural and trunk mobility activities to support overall movement efficiency.

Functional activities were limited to basic, non-progressive tasks, such as reaching, lifting light objects, and gross grasp-and-release movements, primarily involving power grips. Importantly, the control program did not include structured or progressive training of MD, precision grips, thumb opposition, coordinated finger movements, or small-object manipulation. Task complexity was adjusted only for safety and tolerance, without systematic progression or individualization targeting fine motor performance.

## 3. Results

All statistical analyses were performed using Python (SciPy and Pandas 2.3.3 libraries). For each outcome (Kapandji, CRT, and PPT subtests), change scores were computed as Δ = (final score − initial score) for every participant. Between-group differences in change scores were then tested using one-way analysis of variance (ANOVA) with group allocation (EG vs. CG) as the independent factor and Δ as the dependent variable. The F-statistic was calculated as the ratio of mean square between groups to mean square within groups, and *p*-values were derived from the corresponding F-distribution.

To quantify the magnitude of between-group effects, eta squared (η^2^) was computed for each outcome as η^2^ = SS_between/SS_total, where SS_between represents the between-group sum of squares based on deviations of group means from the grand mean, and SS_total represents the total sum of squares based on deviations of individual scores from the grand mean. Results are reported as mean ± standard deviation (SD), with statistical significance set at *p* < 0.05.

One-way analysis of variance was performed to evaluate between-group differences in functional outcomes, using the change scores (Δ = final − initial) for each test as dependent variables and group allocation (EG vs. CG) as the independent factor. The analysis revealed a significant main effect of group for most measures of MD and fine motor performance. Specifically, participants in the EG demonstrated significantly greater improvements in thumb opposition, as assessed by the Kapandji scale (*F*(1,28) = 46.67, *p* < 0.001, η^2^ = 0.63), compared with the CG, indicating a marked enhancement of prehension capacity following the targeted rehabilitation program (Table 1).

Similarly, significant between-group differences were observed for coin rotation performance in both the dominant and non-dominant hands. Improvements in coin rotation speed were significantly higher in the EG for the right hand (*F*(1,28) = 62.01, *p* < 0.001, η^2^ = 0.69) and the left hand (*F*(1,28) = 90.60, *p* < 0.001, η^2^ = 0.76), reflecting substantial gains in psychomotor processing speed and digital coordination (Table 1).

Analysis of the PPT further confirmed the superiority of the experimental intervention. One-way ANOVA indicated significant group effects for single-hand performance with the left hand (*F*(1,28) = 35.92, *p* < 0.001, η^2^ = 0.56) and the right hand (*F*(1,28) = 45.15, *p* < 0.001, η^2^ = 0.62), as well as for bilateral coordination (*F*(1,28) = 22.62, *p* < 0.001, η^2^ = 0.45). These findings suggest that the experimental rehabilitation program effectively enhanced fine MD and bimanual integration beyond the effects of standard PT.

In contrast, no statistically significant between-group difference was observed for the PPT subtest (*F*(1,28) = 1.47, *p* = 0.235, η^2^ = 0.05), indicating that complex sequential assembly skills were not substantially modified over the intervention period.

Overall, the effect sizes associated with the significant outcomes were large to very large, with η^2^ values ranging from 0.45 to 0.76, demonstrating that group allocation accounted for a substantial proportion of variance in functional improvement. These results underscore the effectiveness of the targeted MD rehabilitation program in improving upper limb function compared with conventional PT alone.

Following the ANOVA results summarized in Table 1, graphical analyses illustrate the pattern of changes observed within and between groups.

**Figure 1:**
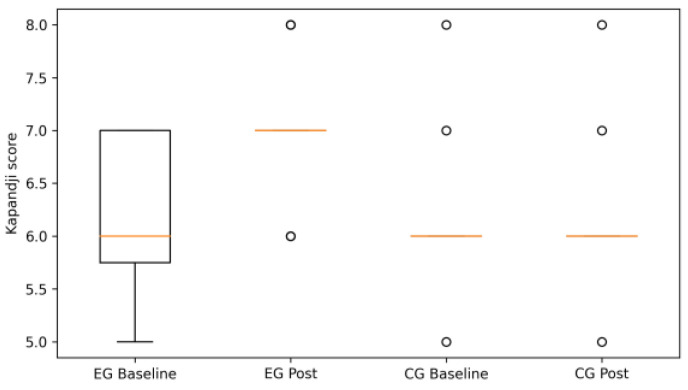
Kapandji scores at baseline and post-intervention in the experimental (EG) and control (CG) groups (box plots: median, interquartile range, spread; outliers shown as points).

Improvements in thumb opposition were observed predominantly in the EG, as reflected by higher post-intervention Kapandji scores, whereas the CG showed no meaningful change (Figure 1). The separation between baseline and post-intervention values in the EG is consistent with the large between-group effect identified in the change score analysis.

**Figure 2:**
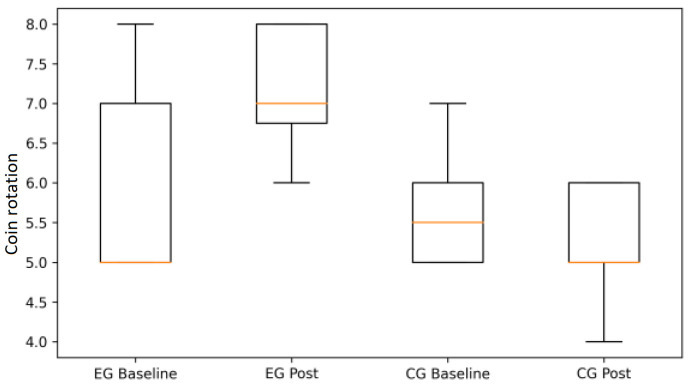
Coin rotation performance (dominant/right hand) at baseline and post-intervention in the experimental (EG) and control groups (CG) (box plots).

**Figure 3:**
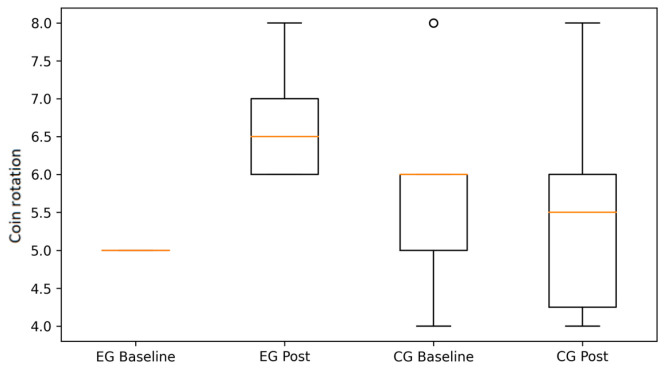
Coin rotation performance (non-dominant/left hand) at baseline and post-intervention in the experimental (EG) and control groups (CG) (box plots). Boxes represent the interquartile range (IQR), the horizontal line indicates the median, whiskers denote minimum and maximum values, and circles represent individual outliers.

Coin rotation performance exhibited a similar pattern. Participants in the EG demonstrated clear improvements in both dominant and non-dominant hands, while the CG showed stable or declining performance over time (Figure 2 and Figure 3). These findings visually support the significant between-group differences in psychomotor speed and digital coordination reported in Table 1.

**Figure 4:**
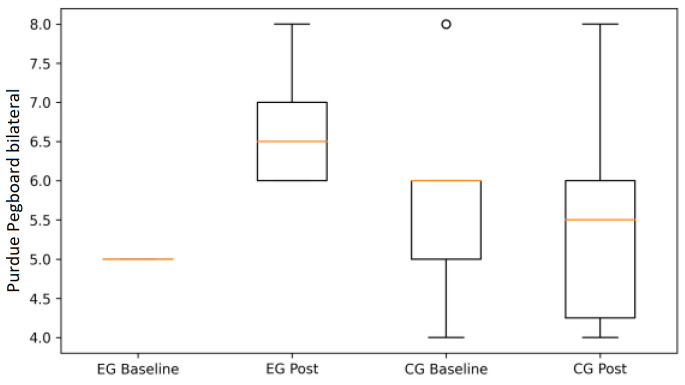
Purdue Pegboard bilateral performance at baseline and post-intervention in the experimental (EG) and control groups (CG) (box plots). Boxes represent the interquartile range (IQR), the horizontal line indicates the median, whiskers denote minimum and maximum values, and circles represent individual outliers.

**Figure 5:**
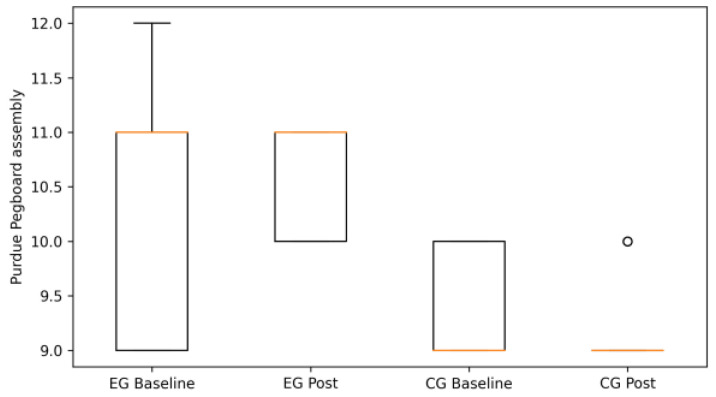
Purdue Pegboard Assembly task performance at baseline and post-intervention in the experimental (EG) and control groups (CG) (box plots). Boxes represent the interquartile range (IQR), the horizontal line indicates the median, whiskers denote minimum and maximum values, and circles represent individual outliers.

**Figure 6:**
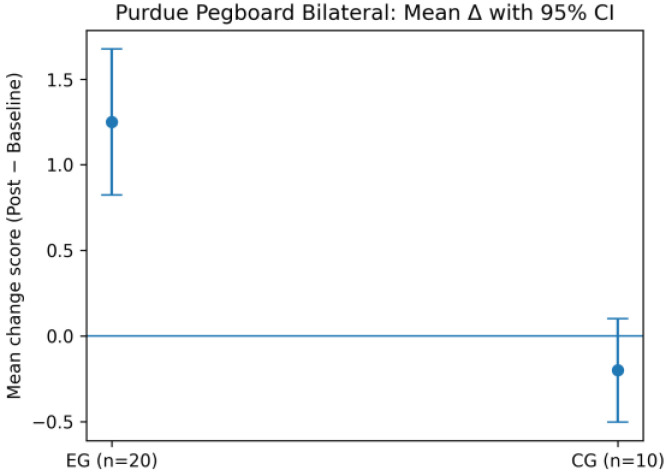
Mean change (Δ = post − baseline) in Purdue Pegboard bilateral scores with 95% confidence intervals for both groups.

For the PPT, the EG showed marked gains in unilateral (left and right hand) and bilateral tasks, indicating enhanced fine motor dexterity and interlimb coordination (Figure 4, Figure 5 and Figure 6). In contrast, the CG exhibited minimal change or slight deterioration across these measures, in line with the direction and magnitude of the observed change scores.

**Figure 7:**
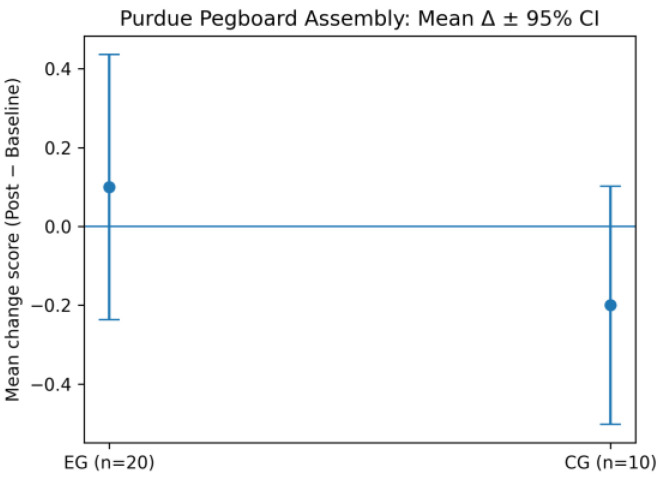
Mean change (Δ = post − baseline) in Purdue Pegboard assembly scores with 95% confidence intervals for both groups.

To further illustrate the between-group differences identified by the ANOVA, bilateral and assembly performance are presented as mean change scores (Δ = post − baseline) with 95% confidence intervals (Figure 6 and Figure 7).

For bilateral PPT performance, the EG shows a positive mean change with confidence intervals not overlapping zero, whereas the CG demonstrates minimal change. In contrast, for the PPT Assembly task, confidence intervals overlap zero in both groups, confirming the absence of a statistically significant intervention effect and supporting the results reported in Table 1.

## 4. Discussion

The findings of the present study should be interpreted within the context of the pragmatic clinical design. Comparable baseline characteristics and diagnostic distribution support the attribution of functional improvements primarily to the targeted MD intervention. However, given the neuropsychiatric complexity of PD and PS, medication-related motor effects cannot be entirely excluded.

All participants were assessed under stable pharmacological conditions, reflecting routine clinical practice. Despite potential confounding from dopaminergic or antipsychotic-related motor effects, the consistent superiority of the EG across multiple dexterity outcomes suggests that structured, task-specific rehabilitation induces meaningful gains beyond pharmacological treatment alone. The absence of improvement in the assembly task indicates that complex sequencing abilities may require longer interventions or explicit cognitive–motor training components, particularly in populations with executive dysfunction.

The present study demonstrates that a structured, progressive PT program incorporating targeted MD training produces significant functional benefits in patients with PD and PS. Compared with conventional PT, the experimental intervention resulted in superior improvements in thumb opposition, psychomotor processing speed, and unilateral and bilateral fine motor performance.

Improvements observed in the Kapandji opposition score highlight the effectiveness of targeted thumb-focused interventions in enhancing prehension capacity. Given the central biomechanical role of the thumb in both power and precision grips, these findings are clinically meaningful, particularly in populations affected by bradykinesia, rigidity, and psychomotor slowing. The absence of improvement in the CG suggests that standard PT alone may be insufficient to address fine motor deficits [45,46,47].

Similarly, significant gains in CRT performance indicate enhanced psychomotor processing speed and digital coordination in the EG. This is particularly relevant in PD, where bradykinesia and movement initiation deficits are prominent, and in PS, where psychomotor slowing and impaired coordination are common. The sensitivity of the CRT to change supports its utility as both an assessment and therapeutic tool in rehabilitation settings [41,42,43,44].

The PPT findings further corroborate the superiority of the targeted intervention, with marked improvements in unilateral and bilateral tasks reflecting enhanced fine motor dexterity and interlimb coordination. In contrast, the lack of significant change in the assembly subtest suggests that complex sequential motor planning may require longer intervention periods or additional cognitive–motor training components, especially in older adults with cognitive comorbidities [38,39,40].

The large effect sizes observed across most outcomes underscore the clinical relevance of integrating dexterity-specific training into rehabilitation programs. Importantly, the comparable baseline characteristics between groups, including age, diagnosis, and comorbidities, support the validity of attributing functional improvements to the intervention rather than to spontaneous recovery or disease variability.

PD is a progressive neurodegenerative disorder caused by dopaminergic neuronal loss in the basal ganglia and is characterized by bradykinesia, rigidity, tremor, and postural instability. These motor and cognitive impairments significantly impact ADL, particularly upper extremity functions such as reaching, grasping, and manipulating objects. Therefore, simple and reliable clinical measures are essential for accurately assessing upper limb function and evaluating the effects of therapeutic interventions in patients with PD [48,49].

Hwang et al. examined the relationship between MD and the Unified Parkinson’s Disease Rating Scale—Motor Examination (UPDRS-ME) as measures of upper limb function in patients with PD. Thirty-two individuals with idiopathic PD were assessed using the box-and-block test and UPDRS-ME. DM scores were positively correlated between the more affected and less affected sides and showed a negative correlation with UPDRS-ME motor scores, indicating that greater motor impairment was associated with reduced dexterity. These findings support the use of both the box-and-block test and the UPDRS-ME as complementary clinical tools for quantifying upper limb function and informing rehabilitation planning [50].

Vanbellingen et al. showed that patients with PD commonly present impaired MD, adversely affecting ADLs such as buttoning, handwriting, and object manipulation. In a single-blinded RCT, a task-specific home-based dexterity program (HOMEDEXT) was compared with a conventional TheraBand exercise program. After four weeks, the HOMEDEXT group demonstrated significantly greater improvements in fine motor performance, assessed by the Nine-Hole Peg Test, with benefits extending to dexterity-related ADL. However, these gains were not sustained at follow-up, indicating that continued, task-specific training is required to maintain improvements in fine motor function in PD [51].

Korkmaz et al. investigated the relationship between MD, attention, and working memory in patients with PD across different disease stages. Patients in mid-stage PD (Hoehn and Yahr stages 3–4) demonstrated significantly poorer hand dexterity, attention, and logical memory performance compared with early-stage patients, while digit span performance was similar between groups. MD was significantly associated with both attention and memory measures, and disease stage correlated with dexterity and selected cognitive outcomes. The authors concluded that dexterity impairment in PD is closely linked to cognitive dysfunction and worsens with disease progression, supporting the integration of combined cognitive and dexterity assessments from the early stages of PD within a multidisciplinary rehabilitation approach [52].

Vasu et al. conducted a systematic review to identify optimal PT interventions for improving MD in patients with PD. Across 11 RCTs involving 647 participants, most interventions demonstrated positive effects on hand dexterity, with several studies also reporting improvements in ADLs and upper limb motor performance. However, the overall quality of evidence was limited by a high risk of performance bias and heterogeneity in intervention duration and content. Despite these limitations, the authors concluded that home-based dexterity-focused rehabilitation programs represent a promising approach for enhancing dexterity-related functional abilities in PD [21].

Carvalho et al. evaluated the effects of PNF on functional independence in elderly patients with PD. In a small cohort of five patients with a mean age of over 80 years, functional outcomes were assessed using the Functional Independence Measure (FIM) before and after a 10-session intervention. Although descriptive improvements in functional patterns, including locomotion and sphincter control, were reported, no statistically significant changes were observed in motor or cognitive independence scores. The authors concluded that while PNF may subjectively enhance certain functional aspects, its effects on overall functional independence remain limited, particularly in advanced-age PD populations [53].

Although cognitive impairments predominate in schizophrenia, sensorimotor abnormalities have been recognized since the earliest descriptions of the disorder [54]. However, their clinical relevance remains debated, particularly regarding whether these deficits are medication-induced, reflect a primary pathophysiological impairment, or could serve as reliable clinical markers of schizophrenia [55,56]. While extrapyramidal symptoms and upper limb motor deficits have traditionally been attributed to antipsychotic treatment, growing evidence suggests that medication alone does not fully account for sensorimotor dysfunction. Instead, motor abnormalities may be linked to altered cognitive processes, such as impaired action planning [57].

Importantly, studies involving drug-naïve or differentially medicated patients have demonstrated genuine sensorimotor deficits, including psychomotor signs, neurological soft signs, postural instability, abnormal eye movements, and impaired upper limb control. These findings support the notion that sensorimotor dysfunction may represent a core feature of schizophrenia or vary with disease state. Consequently, quantitative assessment of motor and MD impairments may have clinical utility as markers of vulnerability or neurodevelopmental abnormalities; however, further validation regarding sensitivity, specificity, and responsiveness is still required [58,59].

Känel et al. investigated motor control impairments in a large cohort of patients with schizophrenia spectrum disorders, focusing on MD and grip force. Using standardized motor rating scales, the authors showed that performance on the CRT and grip force measures was significantly associated with multiple motor abnormalities, with the strongest relationships observed for neurological soft signs (NSSs). Regression analyses demonstrated that NSSs were better predictors of fine motor performance than psychomotor slowing, Parkinsonism, or catatonia. Specifically, deficits in motor coordination and sequencing predicted coin rotation performance, whereas sensory integration was more strongly related to grip force. These findings highlight NSSs as key contributors to impaired MD and support the need for targeted motor interventions in schizophrenia [60].

Emerging evidence links psychomotor impairment across neurological and psychiatric disorders to altered brain-wide regulatory systems, including the glymphatic pathway. Reduced glymphatic clearance, assessed using the DTI-derived ALPS index, has been reported during acute psychosis, suggesting disrupted cerebrospinal fluid dynamics [61]. Although observed in acute settings, such mechanisms may contribute to persistent psychomotor slowing and manual dexterity deficits in stabilized schizophrenia. Consequently, the functional gains observed following targeted manual dexterity rehabilitation may reflect not only task-specific motor learning but also activity-dependent modulation of central neurophysiological processes [61].

Liu et al. examined dual-task effects on hand MD and their relationship with daily functioning in individuals with chronic schizophrenia compared with healthy controls. Performance on hand dexterity tasks declined under dual-task conditions in both groups; however, the reduction was significantly greater in the schizophrenia group. Poorer dual-task dexterity performance was associated with lower ADL scores, indicating a strong link between motor–cognitive interference and functional impairment. These findings suggest that deficits in dual-task MD are a key feature of schizophrenia and support the use of dual-task–based therapeutic approaches to enhance real-world functional abilities [62].

Several limitations should be acknowledged. The exploratory, non-randomized design may introduce selection bias, although baseline comparability mitigates this concern. Detailed medication stratification, including dopaminergic state, antipsychotic class or dose, and extrapyramidal symptom assessment, was not systematically collected and may represent a confounding factor. Additionally, the small sample size limited stratified or sensitivity analyses by diagnosis or baseline severity. Patient-centered outcomes (e.g., ADLs or quality of life) and post-intervention follow-up were not included, limiting assessment of real-world relevance and durability.

Another important limitation of this study is the relatively small sample size, particularly in the CG (*n* = 10), which may have reduced statistical power and limited the detection of smaller between-group effects. Consequently, the results should be interpreted with caution. To mitigate this limitation, effect sizes (η^2^) were reported alongside *p*-values to provide a more informative estimate of the magnitude and clinical relevance of the observed effects. Future studies with larger, adequately powered cohorts are warranted to confirm these findings.

In addition, the absence of long-term follow-up limits conclusions regarding the retention of training effects, and although standard disease-specific PT was provided to the CG, its initial description was limited and may affect intervention comparability.

The generalizability of the present findings should be interpreted cautiously, as the study population comprised clinically stable patients with PD at Hoehn and Yahr stages II–III. Consequently, the results are most applicable to individuals with moderate disease severity and preserved cognitive function, and may not directly extend to early-stage or advanced Parkinsonian syndromes.

Similarly to our study, Vanbellingen et al. (2017) demonstrated that task-specific dexterity training leads to significant improvements in fine motor performance in patients with Parkinson’s disease [51]. However, in contrast to our long-term, supervised intervention, their shorter home-based program showed limited retention at follow-up, underscoring the importance of intervention duration and structured progression for sustained benefits.

In line with our findings, Hwang et al. reported that reduced MD is closely associated with motor impairment severity as assessed by the UPDRS, supporting the clinical relevance of dexterity-focused outcome measures [49,50]. Furthermore, Korkmaz et al. demonstrated that MD impairments worsen with disease progression and are strongly linked to attentional and executive deficits, which may partly explain the lack of improvement observed in complex sequential tasks such as the PPT Assembly in the present study [52]. Finally, the systematic review by Vasu et al. highlighted the overall effectiveness of dexterity-oriented physiotherapy in Parkinson’s disease, while emphasizing heterogeneity in intervention content and duration, a limitation addressed in the structured, phased design of our rehabilitation program [21].

## 5. Conclusions

This prospective interventional study demonstrates that a structured, progressive PT program incorporating targeted MD training produces significant functional benefits in patients with PD and PS. Compared with conventional PT, the experimental intervention resulted in markedly greater improvements in thumb opposition, psychomotor processing speed, and unilateral and bilateral fine motor performance, with large to very large effect sizes.

The findings confirm that MD impairments are modifiable, even in older patients with chronic neurological and psychiatric conditions, when rehabilitation programs specifically address fine motor control, thumb function, and coordinated hand movements. In contrast, the absence of meaningful improvement in the CG highlights the limitations of standard physiotherapy approaches that do not explicitly target dexterity.

Hand functionality represents a key determinant of functional independence and plays a central role in preventing and slowing functional decline in patients with PD and PS. Targeted hand-focused rehabilitation supports sensorimotor integration and neuroplasticity, contributing to the preservation of autonomy, self-care capacity, and social participation. Therefore, hand function should be regarded not as a peripheral outcome, but as a core mediator of global functional status, justifying its systematic inclusion in multidisciplinary rehabilitation strategies for these populations.

The lack of significant change in complex sequential assembly tasks suggests that higher-order motor planning and cognitive–motor integration may require longer intervention durations or additional cognitive–motor training components, particularly in populations with cognitive comorbidities.

Overall, the results support the integration of dexterity-oriented rehabilitation strategies into routine PT programs in residential and outpatient care settings. Incorporating standardized dexterity assessments such as the Kapandji scale, CCR, and PPT allows for sensitive monitoring of functional change and more precise intervention planning.

Future studies should include larger samples, long-term follow-up, and disease-specific subgroup analyses, as well as explore the added value of dual-task and cognitive–motor training, to further optimize rehabilitation outcomes in PD and PS.

## Figures and Tables

**Table 1:** One-way ANOVA on change scores (Δ final − initial).

Test	*n* GE	Δ GE Mean ± SD	*n* CG	Δ CG Mean ± SD	F	*p*	η^2^
Kapandji	20	1.00 ± 0.46	10	0.00 ± 0.00	46.67	<0.001	0.63
Coin Rotation—Right	20	1.10 ± 0.45	10	−0.30 ± 0.48	62.01	<0.001	0.69
Coin Rotation—Left	20	1.15 ± 0.37	10	−0.40 ± 0.52	90.60	<0.001	0.76
Purdue Pegboard—Left	20	1.25 ± 0.79	10	−0.40 ± 0.52	35.92	<0.001	0.56
Purdue Pegboard—Right	20	1.65 ± 0.75	10	−0.20 ± 0.63	45.15	<0.001	0.62
Purdue Pegboard—Bilateral	20	1.25 ± 0.91	10	−0.20 ± 0.42	22.62	<0.001	0.45
Purdue Pegboard—Assembly	20	0.10 ± 0.72	10	−0.20 ± 0.42	1.47	0.235	0.05

Note: η^2^ = effect size (0.01 small, 0.06 medium, ≥0.14 large).

## Data Availability

The original contributions presented in this study are included in the article. Further inquiries can be directed to the corresponding author.

## Abbreviations

The following abbreviations are used in this manuscript:

ADL	Activities of daily living
CG	Control group
CRT	Coin Rotation Task
EG	Experimental group
MD	Manual dexterity
PD	Parkinson’s disease
PNF	Proprioceptive Neuromuscular Facilitation
PPT	Purdue Pegboard Test
PT	Physiotherapy
PS	Paranoid schizophrenia
ROM	Range of motion
